# Femoral neck fracture in sickle cell anemia: A comprehensive case study

**DOI:** 10.1002/ccr3.9413

**Published:** 2024-09-03

**Authors:** Ayman Taj Elsir Mustafa Babiker, Yousif Omer Elgaili Yousif, Mohammed Mubarak Mohammed Ahmed, Mohammed Alssir MohammedAhmed, Hozifa Mohammed Ali Abd‐Elmaged

**Affiliations:** ^1^ Orthopaedics and Traumatology Atbara Teaching Hospital Atbara Sudan; ^2^ General Surgery Department Alzaim Alazhari University Khartoum Sudan; ^3^ Orthopaedics and Traumatology Royal Care International Hospital Khartoum Sudan; ^4^ Orthopaedics and Traumatology Ibrahiem Malik Teaching Hospital Khartoum Sudan; ^5^ Orthopaedics and Traumatology Alzaiem Alazhari University Khartoum Sudan

**Keywords:** dynamic hip screw, femoral neck fracture, hemoglobin sickle cell, postoperative care, stress fracture

## Abstract

**Key Clinical Message:**

The significance of taking femoral neck stress fractures into account as a possible consequence in sickle cell anemia patients is underscored by this case report. In this high‐risk group, early identification, timely diagnosis, and suitable care are crucial for averting major problems and improving results. When sickle cell anemia patients complain with hip or groin discomfort, healthcare practitioners should keep a high index of suspicion for femoral neck stress fractures to assure prompt management and prevent long‐term impairment.

**Abstract:**

Sickle cell disease (SCD) is a prevalent genetic hemoglobinopathy with significant global implications, affecting a substantial portion of the population. Avascular necrosis of the femoral head is a common complication in SCD, leading to severe joint damage and immobility. This case report is of a 20‐year‐old male who presented with severe hip pain due to a femoral neck stress fracture, which progressed to a complete fracture. This patient had a past medical history of sickle cell anemia and a malunited subtrochanteric fracture that resulted in shortening, external rotation, and a limping gait. Surgical treatment via subtrochanteric osteotomy with fixation using a dynamic hip screw with or without iliac crest bone graft was planned, with a satisfactory reduction and closure of the fracture gap, postoperatively. In cases of sickle cell anemia patients, bone complications such as both osteonecrosis and stress fractures are common. As a healthcare provider, it is important to manage and address these not only through medical interventions, but also through counseling and patient education. Patients must be reminded about the importance of compliance with medical advice to avoid progression or recurrence of complications.

## 1 INTRODUCTION

Sickle cell disease (SCD) is a genetic hemoglobinopathy inherited as an autosomal recessive trait with difficulty in hemoglobin synthesis,^1^ leading to a substantial global burden affecting approximately 2%–17% of the population.^2^ Osteonecrosis of the femoral head can manifest at an early age, with some individuals remaining asymptomatic despite joint morphological changes. In severe cases, avascular necrosis (AVN) can progress to destructive osteoarthritis (OA) and joint immobility.^3^ Stress fractures of the femoral neck are rare injuries that can have disastrous consequences if not recognized and treated properly. The consequences of nonunion and femoral head osteonecrosis, which may result in permanent impairment, might arise from incomplete fractures that proceed to completion and displacement.^4^ The most common sites for stress fractures are the femoral neck (5%), metatarsals (16%), fibula (16%), tibia (24%), and tarsal navicular (18%).^5^, ^6^

AVN is a multifactorial condition characterized by disrupted blood and oxygen supply to the bone vasculature, leading to trabecular thinning and bone collapse. In SCD, infarction results from red blood cell (RBC) occlusion of the vasculature due to an altered RBC shape, hindering smooth blood flow and promoting vaso‐occlusion. This process can lead to bone marrow occlusion, ischemia, and eventual AVN development, with up to 50% of sickle cell patients experiencing AVN by 35 years of age. However, AVN is rare in sickle cell trait, a milder form of the disease that is typically asymptomatic.^7^, ^8^, ^9^ Patients with SCD who have end‐stage hip OA as a result of symptomatic AVN are now recommended to have total hip replacement (THR). Patients have a higher risk of hip implant failure than people with THA for primary OA because they are younger, active persons at the time of surgery.^10^ In earlier investigations, implant failure rates ranging from 30% to 60% in less than 5 years were documented. High functional demands from patients frequently cause this, which ultimately results in implant failure.^11^ Compared to individuals experiencing THR following primary hip joint OA, people with SCD are more likely to experience postoperative surgical and medical problems.^12^ Fractures including any tension‐sided involvement or more than or equal to 50% femoral neck involvement are considered high‐risk and should be fixed, according to convention.^13^

This case report highlights the increased risk of orthopedic complications in individuals with this condition. This case can provide valuable insights into the challenges of managing bone health in sickle cell patients, as well as the importance of early detection and intervention to prevent serious complications like AVN, emphasizing the challenges faced during treatment in a young African male. Understanding the expected pre‐ and postoperative course, as well as intraoperative considerations, is crucial for the optimal management of such patients.

## 2 CASE HISTORY/EXAMINATION

A 20‐year‐old male, known to have SCD since the age of 8 months, has been on regular follow‐up. The patient presented with severe left hip pain and an inability to bear weight following a simple fall from a standing height. Further history revealed a prolonged history of groin pain that was relieved by rest and analgesia but had been increasing in severity. Despite being informed of a stress fracture on the femoral neck tension side, the patient disregarded medical advice until it progressed to a complete fracture. Additionally, the patient had a history of a left subtrochanteric fracture 8 years prior, which was traditionally treated by a bone setter and resulted in malunion, shortening, external rotation, and a limping gait. All other systems were normal, and the patient had a good nutritional status and family history. Past medical history included multiple hospitalizations, blood transfusions, and the use of folic acid and hydroxyurea tablets.

On examination, the patient appeared well but pale, was not jaundiced, and was vitally stable but in pain. Physical examination revealed external rotation of the left limb, with an apparent shortening of 4.5 cm. The Bryant's Triangle test was positive for below the greater trochanteric shortening of 4 cm. The distal neurovascular examination was intact, and the right lower limb and both upper limbs were normal.

## 3 METHODS (DIFFERENTIAL DIAGNOSIS, INVESTIGATIONS, AND TREATMENT)

Laboratory tests showed a hemoglobin level of 7.2 g/dL, a total white blood cell count of 10,000 cm^3^, a platelet count of 230 × 10^3^/μL, a normal erythrocyte sedimentation rate, C‐reactive protein, renal function tests, and viral screening.

**FIGURE 1:**
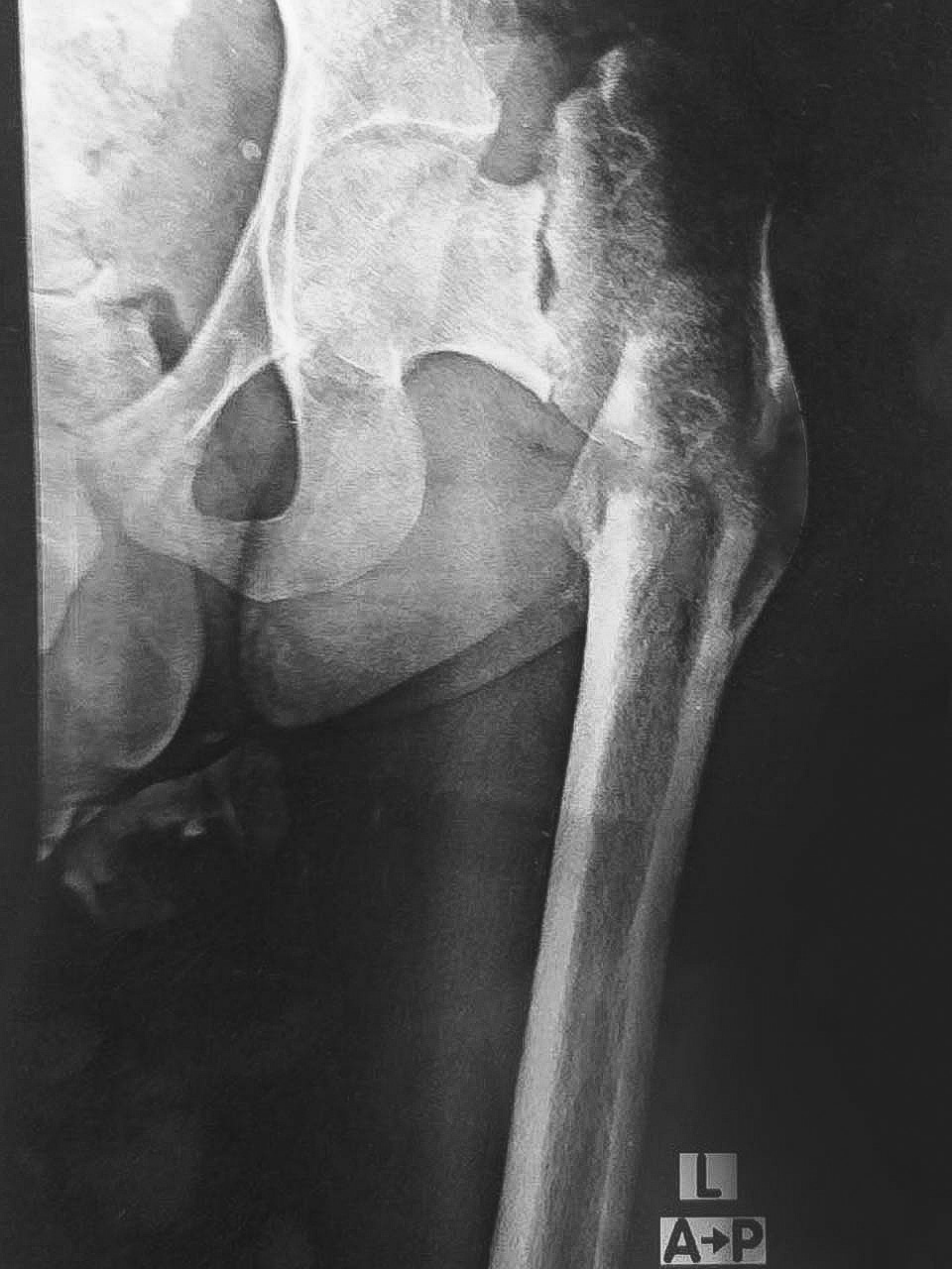
Preoperative X‐ray showing malunited subtrochanteric fracture, basotrochanteric fracture with severe varus and sclerotic fracture ends.

An X‐ray of the left hip revealed a malunited subtrochanteric fracture with severe varus deformity, a neckshaft angle of 85°, a vertically oriented basotrochanteric fracture, and sclerotic fracture ends, indicative of mostly complete fractures on top of old tension‐side stress fractures, as shown in Figure 1. After thorough discussion and counseling with the patient, surgical treatment in the form of a subtrochanteric osteotomy with fixation using a dynamic hip screw with or without an iliac crest bone graft was planned.

The surgery was performed on a traction table under spinal anesthesia with imaging intensifier guidance. A lag screw was positioned centrally in the neck under C‐arm guidance, followed by subtrochanteric osteotomy and fixation with a side plate using five screws. The closure was done in layers with Vicryl sutures, and the skin was closed with clips. The patient had an uneventful recovery period with no postoperative complications.

## 4 CONCLUSION AND RESULTS (OUTCOME AND FOLLOW‐UP)

**FIGURE 2:**
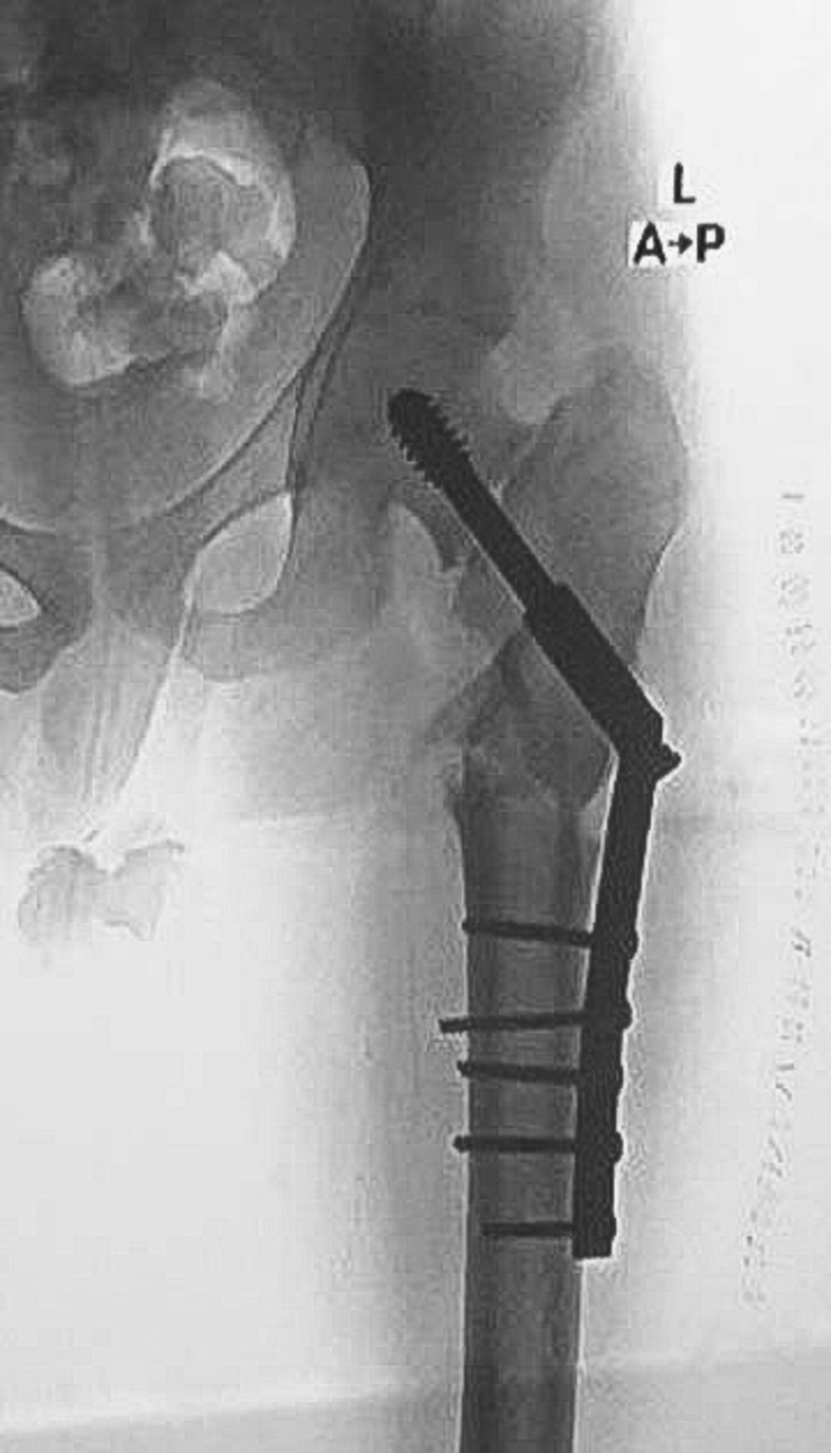
Immediate postoperative X‐ray showing correction of varus neckshaft angle, compression of fracture site, subtrochanteric osteotomy, and fixation with dynamic hip screw.

Postoperatively, immediate X‐rays showed satisfactory reduction and closure of the fracture gap, as shown in Figure 2. The patient was kept non‐weight‐bearing for the first 2 weeks, after which the clips were removed, and gentle range of motion (ROM) exercises at the hip, knee, ankle, and toe were initiated with partial weight‐bearing using a Zimmer frame, gradually progressing to full weight‐bearing at 6 weeks. The rehabilitation protocol included ROM exercises initiated on the first postoperative day for the hip, knee, and ankle joints. Weight‐bearing activities began with toe‐touch weight bearing as tolerated using a Zimmer frame. The weight‐bearing status was gradually progressed to partial weight bearing at 3 weeks, postoperatively. Subsequently, full weight bearing was permitted once callus formation was observed on X‐ray imaging at 6 weeks post‐surgery.

**FIGURE 3:**
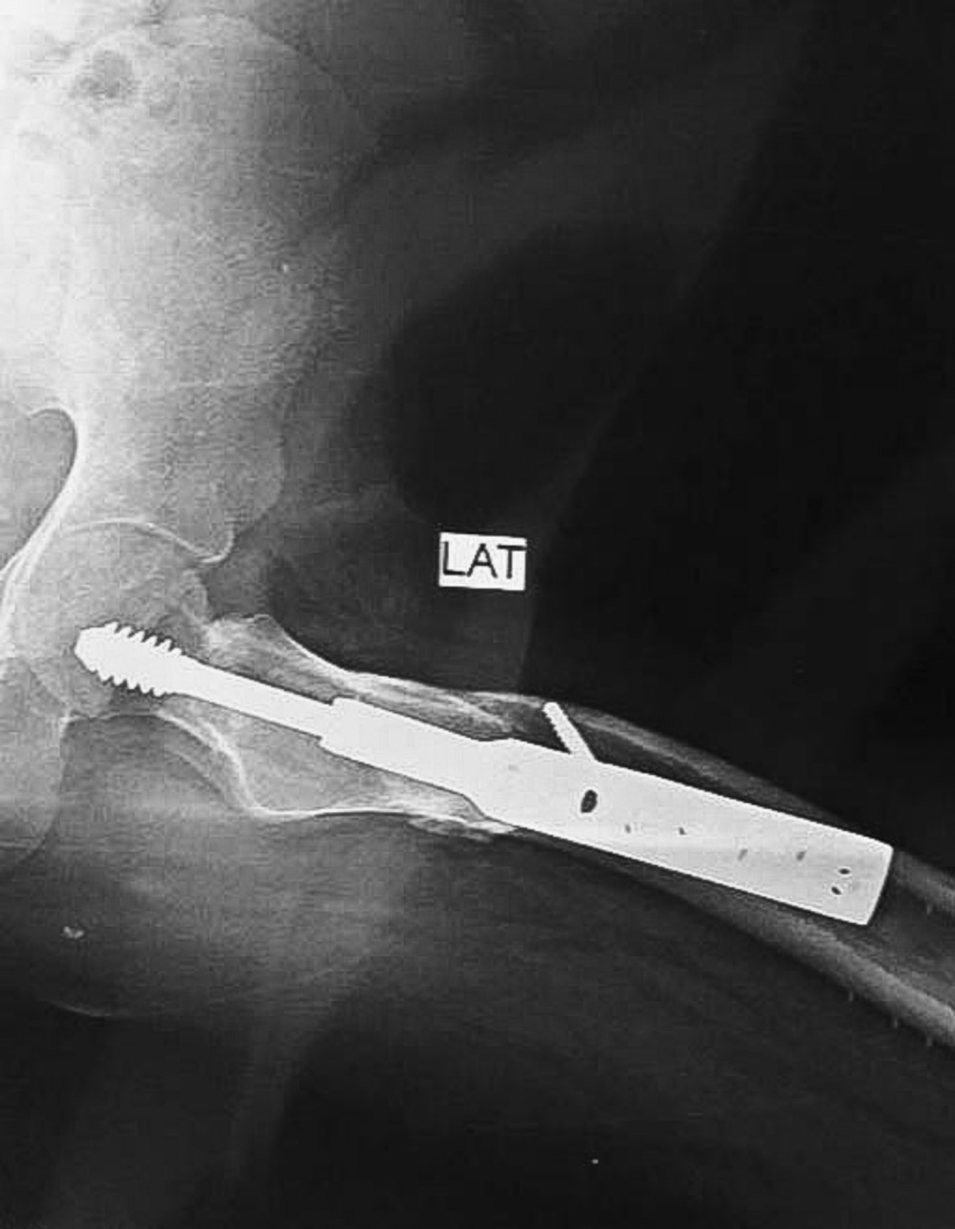
Lateral radiograph of the patient after 2 years, postoperativerly, showing a well healed fracture and osteotomy sites.

**FIGURE 4:**
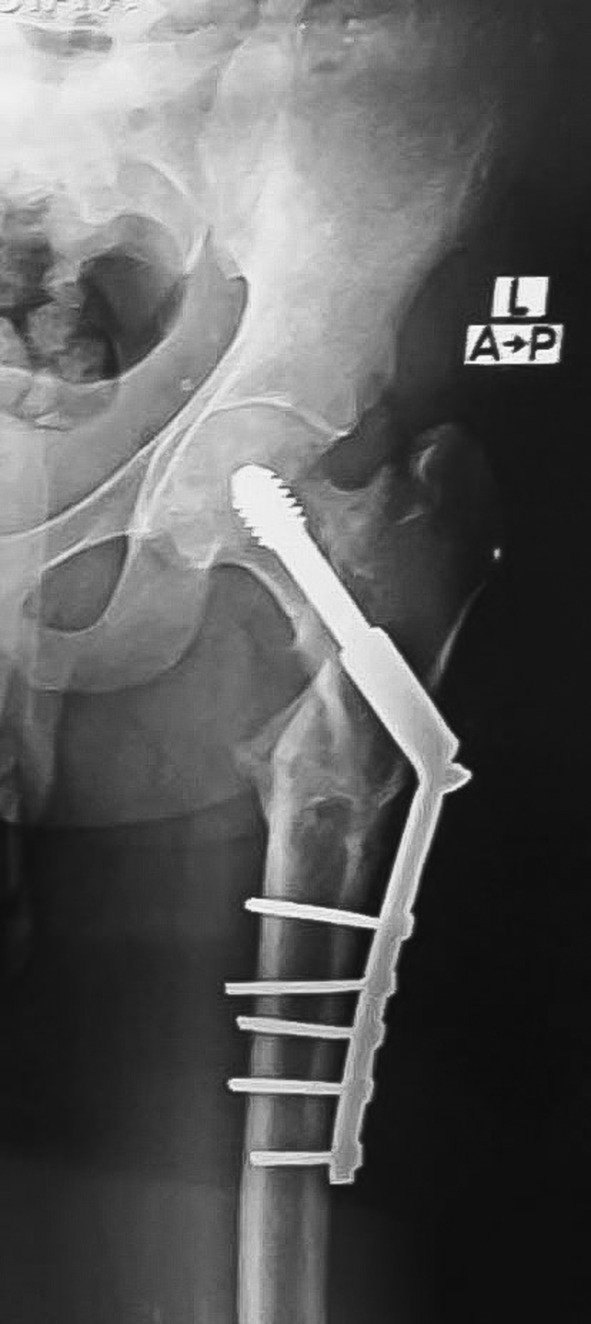
Antero‐posterior radiograph of the same patient after 2 years, post‐operatively, shows a well‐healed fracture and osteotomy sites.

At the 4‐month follow‐up, the patient had a full range of motion at the hip, a well‐healed scar, and no lower limb length discrepancy. After 2 years of follow‐up, the patient remained well and satisfied with no complications noted on X‐ray imaging, as shown in Figures 3 and 4.

The presented case is exceptional due to the presence of a stress fracture with a sclerotic fracture end, complicated by subtrochanteric malunion and varus deformity, in a sickle cell patient not under follow‐up. Remarkably, healing occurred despite the presence of the sclerotic fracture end without the need for a bone graft. This unique case highlights the significance of employing a multidisciplinary approach, conducting comprehensive clinical evaluations, utilizing surgical expertise, and implementing customized rehabilitation strategies when managing femoral neck fractures in individuals with SCD. By acknowledging and addressing the specific challenges associated with hematologic disorders, healthcare professionals can enhance treatment outcomes, facilitate patient recovery, and mitigate the potential complications linked to orthopedic fractures in this particular patient demographic.

## 5 DISCUSSION

Femoral neck stress fractures can be a complication of osteonecrosis of the femoral head in patients with sickle cell anemia.^14^ These fractures are typically categorized into three types: fatigue, insufficiency, and pathological fractures.^15^, ^16^ While femoral neck fractures often result from falls, they can also be linked to chronic stress rather than a single traumatic incident. Osteoporosis is a significant risk factor for these fractures.^17^ In our case, we believe that the insufficiency fracture of the femoral neck was a consequence of extensive involvement of the femoral head by AVN and associated osteoporosis in the femoral neck and bone marrow infarcts. The patient's history of SCD since early childhood, previous subtrochanteric fracture with malunion, and neglect of medical advice regarding the femoral neck stress fracture underscore the challenges in managing orthopedic conditions in individuals with chronic hemolytic disorders. Patient education and compliance play a crucial role in preventing complications and optimizing treatment outcomes. The clinical findings of external rotation, limb shortening, and positive tests for above‐knee and below‐greater trochanteric shortening highlight the importance of a thorough physical examination in assessing fracture deformities and limb abnormalities. Additionally, laboratory tests and imaging studies, including X‐rays revealing malunion and severe varus deformity, aid in characterizing the extent of the fracture and guiding treatment decisions. The decision to proceed with surgical treatment, specifically subtrochanteric osteotomy with fixation using a dynamic hip screw and potential iliac crest bone graft, reflects a comprehensive approach to addressing the complex nature of the femoral neck fracture. Surgical precision, aided by imaging intensifier guidance, is crucial in achieving optimal fracture reduction and stability. The emphasis on postoperative care, including non‐weight bearing status, gradual initiation of ROM exercises, and progressive weight‐bearing protocol, demonstrates the importance of guided rehabilitation in promoting healing and restoring function. Close monitoring and adherence to the prescribed rehabilitation regimen are essential for the patient's successful recovery. The patient's uneventful recovery period and satisfactory closure of the fracture gap on postoperative X‐rays indicate successful surgical intervention and postoperative management. Long‐term follow‐up and monitoring are necessary to evaluate the integrity of the fixation, assess bone healing, and detect any potential complications or recurrent fractures. Collaborative efforts among orthopedic surgeons, hematologists, and other healthcare providers are integral in managing orthopedic complications in sickle cell patients. Patient counseling, education, and shared decision‐making facilitate informed choices regarding treatment options and postoperative care, contributing to a comprehensive and patient‐centered approach.

Sickle cell anemia patients need information about risk for femoral neck stress fractures and the importance of seeking medical help if they have any pain or discomfort in their hip or groin. Physicians should be alert to the possibility of sickle cell anemia causing femoral neck stress fractures, especially active patients with a history of bone pain. Early detection and treatment is critical to avoid complications such as AVN and THR surgery. Aggressive non‐surgical measures including rest, analgesics, and physiotherapy are usually quite effective in managing femoral neck fractures among SCA clients. In some instances however, surgical intervention maybe necessary when the fracture is displaced or fails to heal using conservative treatment.

Other recommendations include conducting a larger study to determine the occurrence, risk factors, and consequences of femoral neck tension breaks in sickle cell anemia patients also comparing the efficacy of diverse treatment options for femoral neck tension breaks in sickle cell anemia patients and investigating the role of bone marrow transplantation in preventing femoral neck stress fractures in sickle cell anemia patients.

The findings in this article may not apply to all people who have sickle cell anemia with femoral neck stress fractures since it is based on one case report from a single patient. The patient in this case report was treated at a single center in Sudan hence the findings may not be generalizable to other centers or any other country.

## AUTHOR CONTRIBUTIONS

**Ayman Taj Elsir Mustafa Babiker:** Conceptualization; project administration; resources; validation; visualization. **Yousif Omer Elgaili Yousif:** Methodology; project administration; resources; validation; visualization; writing – original draft. **Mohammed Alssir Mohammed Ahmed:** Conceptualization; project administration; resources; validation; visualization. **Mohammed Mubarak Mohammed Ahmed:** Conceptualization; project administration; resources; validation; visualization. **Hozifa Mohammed Ali Abd‐Elmaged:** Conceptualization; data curation; project administration; resources; supervision; validation; visualization.

## FUNDING INFORMATION

No funding was obtained.

## CONFLICT OF INTEREST STATEMENT

The authors declare no conflicts of interest.

## ETHICS STATEMENT

Written informed consent was obtained from the patient.

## CONSENT

Written informed consent was obtained from the patient to publish this report in accordance with the journal's patient consent policy.

## Data Availability

Data are available on request from the authors.
